# Etymologia

**DOI:** 10.3201/eid1701.ET1701

**Published:** 2011-01

## Etymologia:* Vibrio vulnificus*

### [vĭb’re-o vŭl-nĭf’ĭ-kəs]

From the Latin *vibrio *(to move) and *vulnificus* (causing wounds). *Vibrio vulnificus *is a virulent, gram-negative, comma-shaped, motile bacterium that belongs to the family Vibrionaceae. In 1976, researchers at the Centers for Disease Control identified it as a *Vibrio* sp. and possible emerging pathogen. Because of its association with blistering skin infections, the bacterium was named *Vibrio vulnificus *in 1979. 

Sources: Farmer JJ III. Vibrio (“Beneckea”) vulnificus, the bacterium associated with sepsis, septicaemia and the sea. Lancet. 1979;2:903. PubMed;Hollis DG, Weaver RE, Baker CN, Thornsberry C. Halophilic Vibrio species isolated from blood cultures. J Clin Microbiol. 1976;3:425–31. PubMed;Todar K. Todar’s online textbook of bacteriology. Vibrio vulnificus. [cited 2010 Nov 24]. http://textbookofbacteriology.net/v.vulnificus.html; Dorland’s illustrated medical dictionary. 31st ed. Philadelphia: Saunders Elsevier; 2007.

